# Nanoarchitectonics of molecular self assembled monolayers by transition metal ion intercalation for enhancement of molecular junction conductivity

**DOI:** 10.1039/d4ra02950j

**Published:** 2024-07-08

**Authors:** Y. Tong, M. Alsalama, G. R. Berdiyorov, Sara Iyad Ahmad, H. Hamoudi

**Affiliations:** a Qatar Environment and Energy Research Institute, Hamad Bin Khalifa University Doha Qatar hhamoudi@hbku.edu.qa hichamhamoudia@gmail.com; b HBKU Core Labs, Hamad Bin Khalifa University Doha Qatar

## Abstract

This research delves into the role of metal ions in enhancing the electronic properties of 5,5′-bis(mercaptomethyl)-2,2′-bipyridine (BPD) self-assembled monolayers (SAMs). It combines experimental techniques and numerical simulations to understand the impact of these ions on the structural, electronic, and transport properties of BPD SAMs. Key findings include the varied bonding preferences of metal ions and their significant role in modifying the electronic structure of BPD molecules, leading to enhanced electron delocalization and migration. The study highlights the potential of metal ions in advancing molecular electronics, particularly in the development of high-performance electronic and energy devices.

## Abstract 2

This research delves into the role of metal ions in enhancing the electronic properties of 5,5′-bis(mercaptomethyl)-2,2′-bipyridine (BPD) self-assembled monolayers (SAMs).

## Graphical abstract

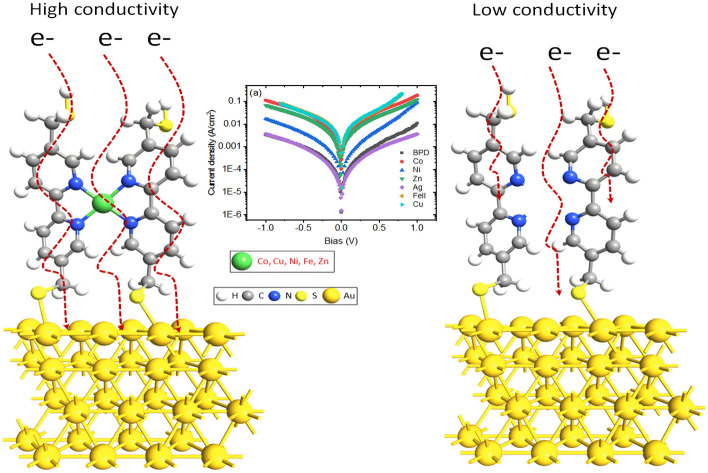

## 1. Introduction

The electronics industry is currently grappling with significant challenges due to the inherent physical limitations of silicon technologies at the nanoscale. Molecular electronics have emerged as a promising solution to bypass these limitations and enhance device performance at sub-nanometer dimensions, leveraging their exceptional functionality at such scales, refer to ref. 1–5 for recent reviews. A key advantage of molecular electronics lies in the vast array of structural and electronic functionalities available, which vary based on the synthesis method employed.^6–10^

The operational characteristics of molecular devices can be fine-tuned by modifying either the molecular backbone or the edge groups that connect to the electrodes, see ref. 11–13 for further details. The strategy of using self-assembled monolayers (SAMs) is particularly effective in molecular electronics, primarily due to the role of the anchoring group. This group is crucial as it determines the strength of the molecule's coupling to the electrode, thereby significantly influencing the structural, electronic, and transport properties of the molecular junctions, see ref. 14–22.

Adjusting the electron distribution within molecules is crucial in the fabrication of molecular devices. Consequently, modifications in the molecular backbone directly impact the device's functional properties. For instance, the transport characteristics of molecular structures can be significantly modified by incorporating resistive or conductive units into the molecular backbone.^23–25^ Moreover, the spatial arrangement of the molecules is also a key factor in determining the operational characteristics of these devices.^26,27^

Another level of complexity and, consequently, functionality is added to the system when redox-active molecules are incorporated into the molecular junctions, see ref. 13 and 28 recent reviews. In these systems, metallic redox components directly contribute to the molecule-electrode coupling by creating additional energy levels accessible for the charge carriers and, consequently, contribute to electronic functionality such as conductance switching and rectification. For example, highly conductive molecular structures can be obtained by incorporating metallic centers into the organic backbone,^29,30^ which contributes to filling the energy gap between the Fermi levels of the electrodes. To achieve desired functionalities in such complex molecular systems, it is important to fully understand the charge transport characteristics taking into account the quantum nature of the charge carriers.^13^

**Fig. 1:**
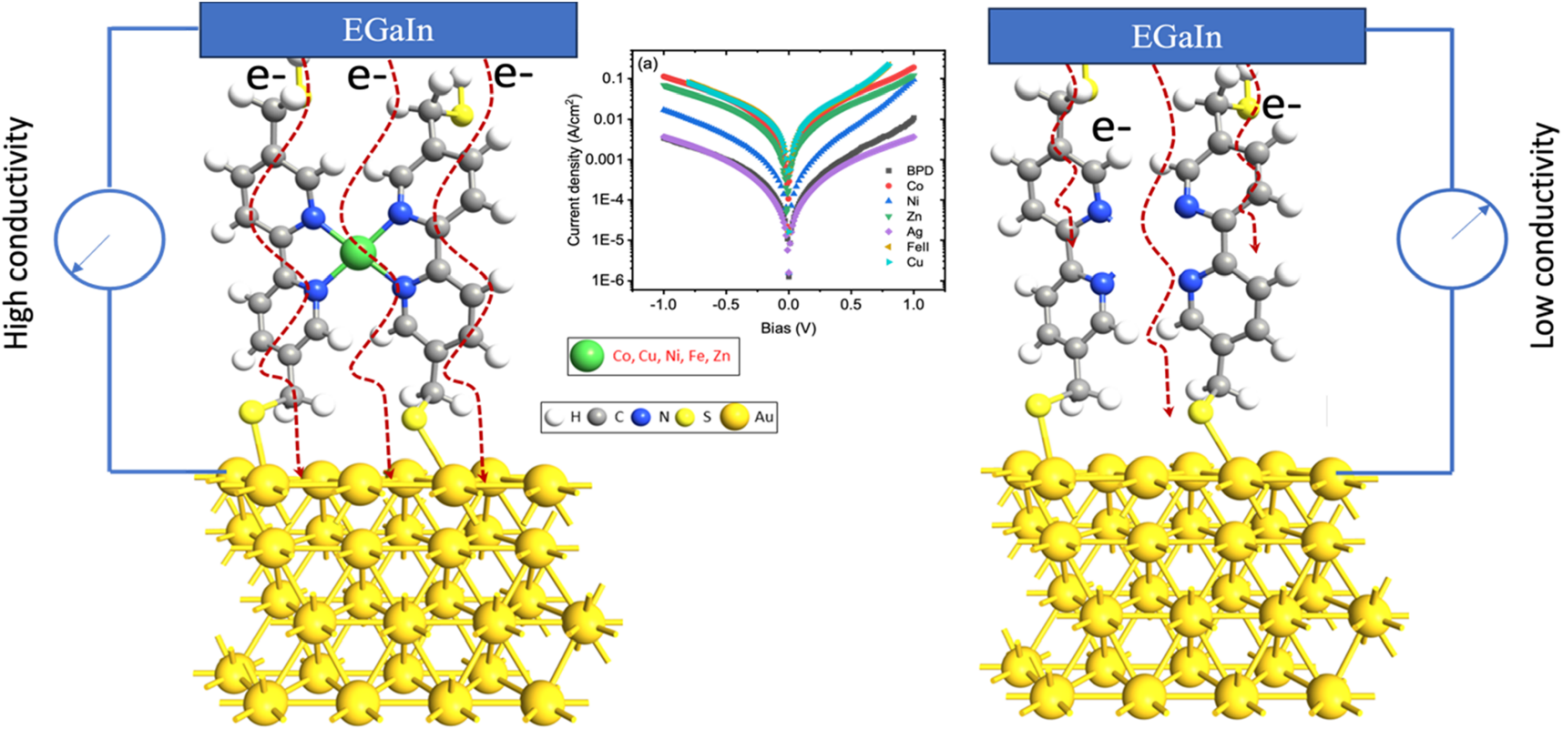
Schematic depiction of the molecular electronics circuit of BPD molecules, both with and without metal ion attachment, on a gold (111) substrate.

In this study, we explore how embedding transition metal ions affects the structure, electronic properties, and transport characteristics of 5,5′-bis(mercaptomethyl)-2,2′-bipyridine (BPD; HS–CH_2_–(C_5_H_3_N)_2_–CH_2_–SH) molecules self-assembled on a gold (111) surface (refer to Fig. 1). The investigation includes both monovalent (Ag^1+^) and divalent (Co^2+^, Cu^2+^, Fe^2+^, Ni^2+^, and Zn^2+^) metal ions. Transport experiments with EGaIn top electrodes reveal a significant increase in current through the molecular SAM in the presence of divalent metal ions. Conversely, only the silver ions lead to a slight decrease in current.

To investigate the reasons behind these conductivity variations based on the ions used, we utilized Ultraviolet Photoelectron Spectroscopy (UPS) and X-ray Photoelectron Spectroscopy (XPS). Analysis of the UPS spectra indicates alterations in the valence band depending on the embedded metal ions, suggesting modifications in the electronic structure of the system. Further, an in-depth examination of the XPS spectra of the molecular self-assembled monolayers reveals that the silver ions predominantly associate with the top thiol units, while the other ions primarily bind to the pyridinic units, exhibiting varying coordination numbers. The obtained results will provide useful information in understanding of the fundamental physics governing the charge transport in molecular devices.^31^

The experimental results are supplemented by spin-dependent quantum transport calculations using the nonequilibrium Green's functional formalism in combination with density functional theory (DFT). We have considered both ferromagnetic (Co, Fe and Ni) and diamagnetic (Ag, Cu and Zn) atoms attached to the pyridinic units of the molecules.^32,33^ The whole molecular system is sandwiched symmetrically between Au (100) electrodes which is the most suitable electrode material for fundamental studies.^12^ We found that regardless of their nature, the presence of the metal atoms increases the electronic transport through the junction considerably due to the structural changes in the backbone of the molecule. In addition, metal atoms create additional energy levels near the Fermi level of the system (as revealed in our molecular projected self-consistent Hamiltonian (MPSH) states and transmission eigenstates analysis) which becomes accessible for the charge carriers at operational bias voltages.

## 2. Results and discussions

### 2.1 Transport measurements

**Fig. 2:**
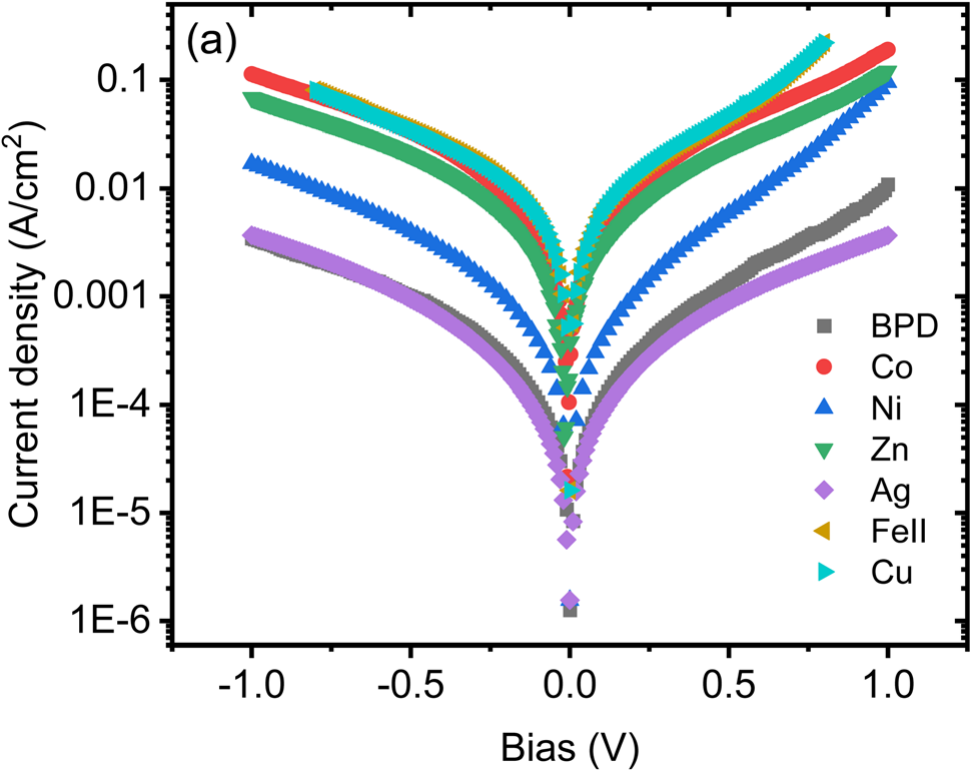
Current–voltage characteristics as a function of bias voltage for BPD SAMs without and with different metal ions.

Fig. 2 displays the recorded current–voltage (*I*–*V*) characteristics for the studied molecular SAMs, both with and without metal ion incorporation. The data shown is the average of 10 successful junctions, with each junction undergoing 10 measurements. From this figure, it is evident that the incorporation of all metal ions leads to an increase in current through the molecular SAM. The highest current is achieved with copper and cobalt ions, while nickel ions also contribute to a higher current, which varies with the applied bias value. In contrast, the silver-BPD setup does not significantly boost the current compared to the other metal ions.

### 2.2 XPS measurements

**Fig. 3:**
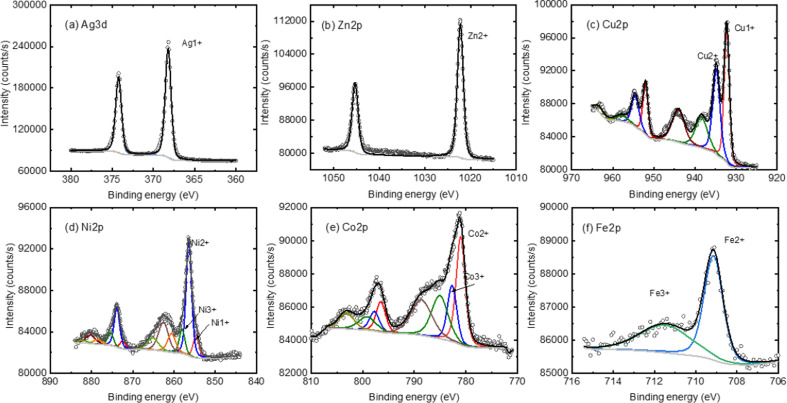
Representative XPS core level spectra of the relative metal ions intercalated in the BPD SAM. The deconvolution is conducted with a Voigt type profile GL(30) after a proper Shirley background subtraction.

XPS spectra of the BPD SAMs are obtained before and after the metal ion embedment. We start with the analysis of the XPS signal corresponding to the metal ions. Fig. 3 shows the XPS spectra of the metals with corresponding fitting curves with a GL(30) profile after a proper Shirley background subtraction. In the case of Ag containing sample, a clear peak corresponding to monovalent silver atom is obtained in the spectrum, see Fig. 3a. A clear XPS signal is also obtained for divalent Zn ion, see Fig. 3b. For all other metal components, XPS peaks corresponding for two different ionic states are obtained: Cu^1+^ and Cu^2+^ ions are detected in the case of copper (Fig. 3c) and metal ions with ionic state of 3^+^ are obtained in all other cases, see Fig. 3d–f.

**Fig. 4:**
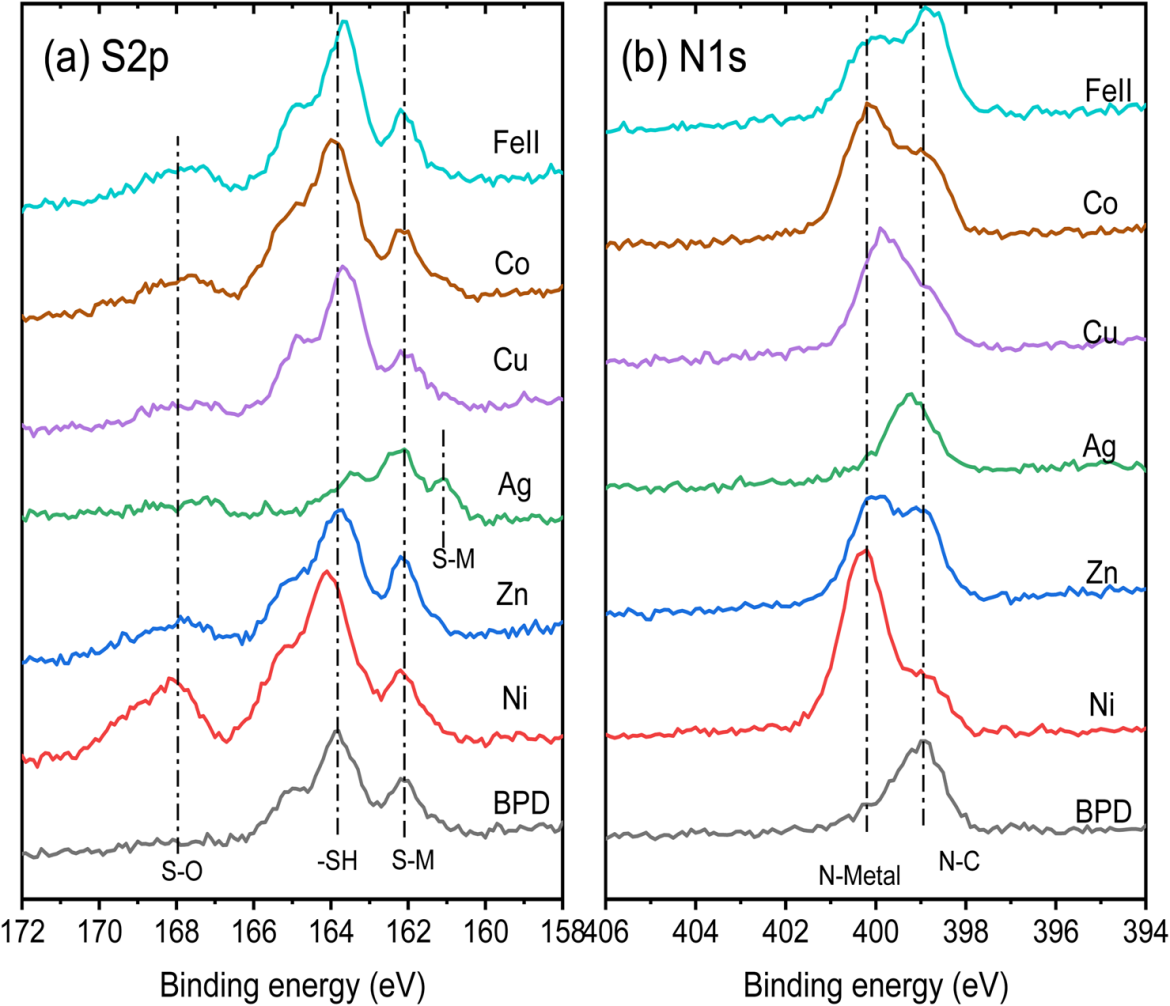
Representative S 2p (a) and N 1s (b) XPS spectra of the considered SAM structures without and with the metal ion embedment.

In general, there are two possibilities of the metal ion attachment to the BPD molecules. The first one is the adsorption of the metal atoms on the top thiol units through sulfur–metal ion covalent bonding and the second is the formation of the covalent bonding between the metal ions and the pyridinic units of the molecule. Fig. 4 compares the S 2p and N 1s core level spectra of all the embedded BPD-SAMs and the corresponding metal-free BPD-SAMs as well, to identify the location of the metal ions. The spectra are shifted vertically for a clear representation. The S 2p signal of the pristine BPD SAM is characterized by two pronounced peaks at 162.1 eV and 163.6 eV (see black curve in Fig. 4a), corresponding to sulfur–metal and free sulfur, respectively. The relative signal amplitudes between the 2 doublets do not change significantly except for silver ions, indicating that the considered metal ions, other than silver, do not prefer to be attached to the top thiol units. The situation is totally different in the case of Ag^1+^ ions: the peak at 163.2 eV disappears and the amplitude of the sulfur signal at 162.1 eV increases considerably. In addition, another component at 161.1 eV newly appears. This indicates that the silver ions are predominantly adsorbed on the top of the SAM with covalent S–Ag bonding. Table 1 shows the details of the S2P spectra analysis where we present the normalized surface areas corresponding to free sulfur and sulfur–metal ion covalent bonding. In the case of pristine BPD SAM, the S–M bonding corresponds to sulfur–gold bonding. The surface area corresponding to the free sulfur does not change considerably for all considered metal ions, except for the silver ions for which the area is reduced by 70%. This indicates that only silver ions prefer to attach to the top of the SAM forming S–Ag covalent bonding.

**Table 1:** Normalized surface areas corresponding to free sulfur, sulfur–metal bonding, free nitrogen, and nitrogen–metal bonding. Calculations are conducted based on 4 different sets of experiments

Samples	S 2p		N 1s	
	Free sulfur (%)	Sulfur–metal (%)	Free nitrogen (%)	Nitrogen–metal (%)
BPD	65.5 ± 4.1	34.5	100	0
BPD + Co	63.1 ± 5.6	31.1	37.2	62.8 ± 6.0
BPD + Ni	60.3 ± 9.6	41.5	42.0	58.0 ± 7.6
BPD + Zn	62.2 ± 3.9	37.7	42.2	55.8 ± 6.3
BPD + Cu	71.1 ± 4.9	27.0	34.7	65.3 ± 13.6
BPD + Ag	16.3 ± 2.9	82.6	100	0
BPD + Fe(ii)	73.8 ± 4.6	30.6	50.7	49.3 ± 12.3

Fig. 4b shows the N 1s core level spectra of the considered samples. For the pristine BPD SAM we obtained a single XPS peak at 399.0 eV which corresponds to N–C covalent bonding. The presence of the metal ions (except Ag ions) reduces the amplitude of this signal and results in the formation of an extra peak at 400.0 eV which can be explained only by the formation of the nitrogen–metal covalent bonding. In the case of silver ions, we did not observe the formation of the second peak in the spectra which indicates weak interactions on the silver ions with the pyridinic units. A small shift of the peak position of the XSP signal can be related to the tilting of the molecules during the silver ions embedment processes in the solution. The coverage percentage of the pyridinic units by the metal ions can be seen in Table 1. The largest coverage is obtained for the Cu ions, whereas Zn and Fe(ii) ions give almost 50% coverage. The results in Table 1 illustrate the robust interaction between metal ions and the nitrogen atoms in the molecular backbone. This interaction causes significant electronic distortion in the BPD molecules. The altered configuration, driven by the metal ions, facilitates additional electron migration channels within the molecular system by increasing the electron delocalization in the molecules.

### 2.3 UPS measurements

**Fig. 5:**
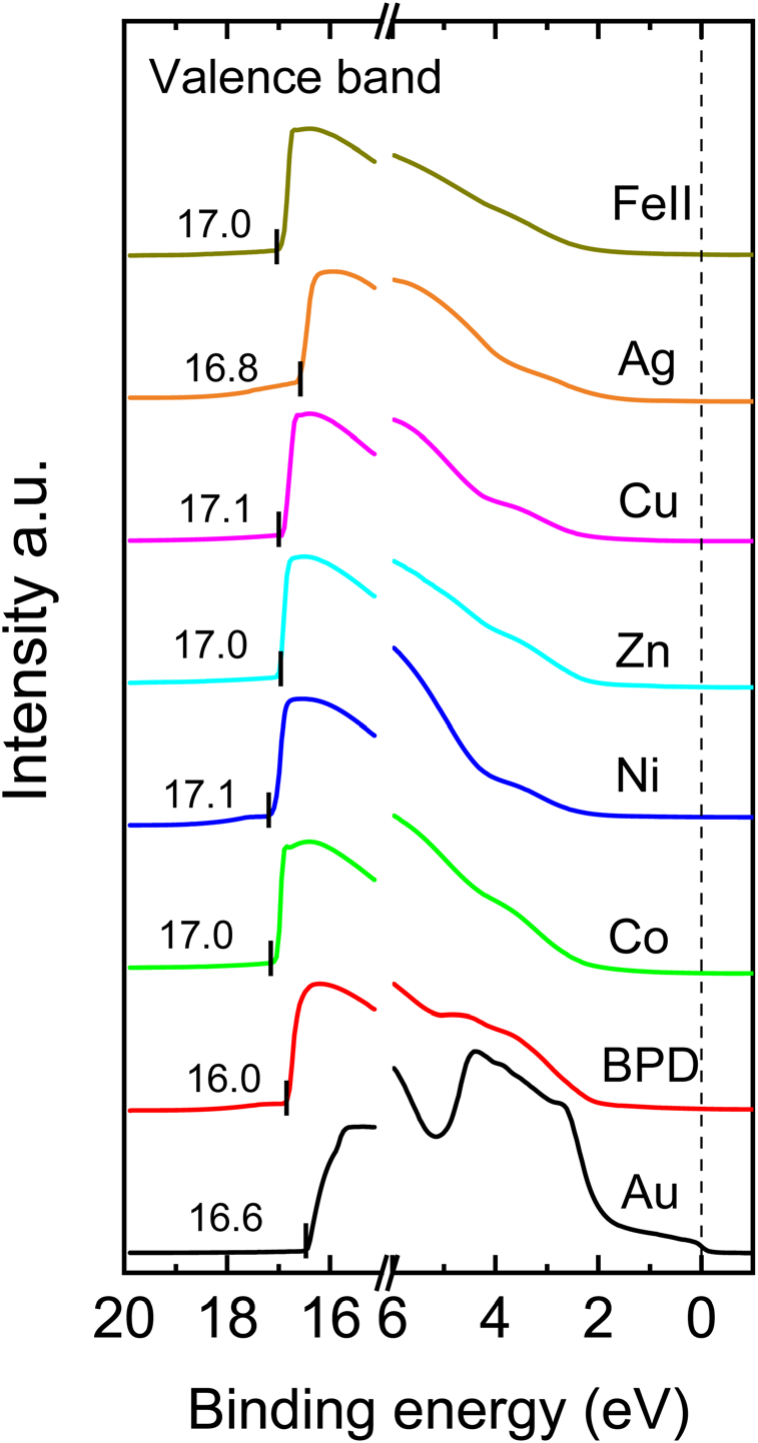
UPS spectra of PBD SAMs on gold substrate for different metal-ion embedment.

Next, we analyze the UPS spectra of the considered samples to study the effect of metal ion embedment on the work function of the system. Fig. 5 shows the UPS spectra of the considered samples. We first identified the Fermi levels of the samples as indicated with numbers to the right of the figure and the cutoff edge at the left side. The difference between these two energy values provides the work function of the molecular SAM on the metal substrate, considering the HeI as the UV source with an energy of 21.22 eV. Table 2 shows the calculated work functions for all considered systems. It is seen that the presence of the metal ions reduces the work function variation of the system. The smallest reduction is obtained for the silver ions whereas the iron ions give the largest reduction of the work function.

**Table 2:** Calculated average work function of the considered systems, based on 4 sets of experiments

SAMs	Work function (eV)
Au	4.71
BPD	4.31 ± 0.15
Co	4.09 ± 0.14
Zn	4.16 ± 0.05
Ni	4.02 ± 0.04
Ag	4.54 ± 0.02
Cu	4.31 ± 0.12
Fe	4.18 ± 0.04

### 2.4 Transport calculations

**Fig. 6:**
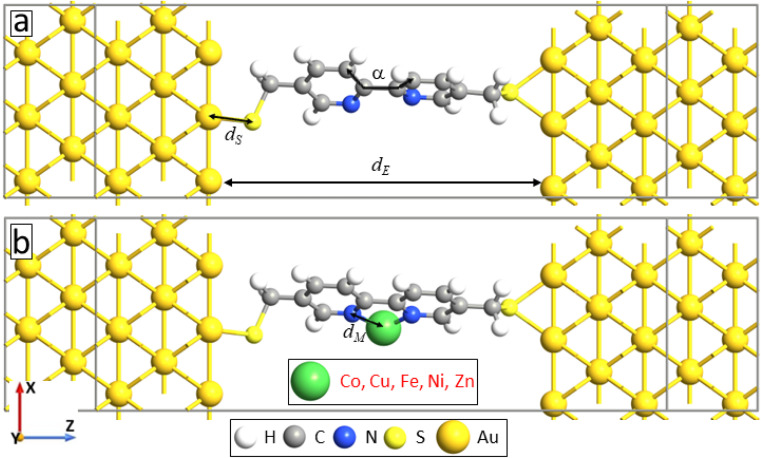
Device geometries: 5,5′-bis(mercaptomethyl)-2,2′-bipyridine molecule sandwiched between the gold electrodes without (a) and with (b) metal atom attachment to the pyridine units (M = Co, Cu Fe, Ni and Zn).

To explain the experimental findings on the effect of metal ions embedment on the transport properties of molecular junctions, we have conducted quantum transport calculations for device geometries consist of 5,5′-bis(mercaptomethyl)-2,2′-bipyridine molecule sandwiched between gold (100) electrodes through S–Au covalent bonding without and with metal atoms attached to the pyridinic units, see Fig. 6a and b, respectively. Note that the sulfhydryl group is deprotonated to make stronger bond with the gold electrode.^11^ Both right and left electrodes are modeled as a semi-infinite extension of the gold supercell with size 7.06 Å.

**Fig. 7:**
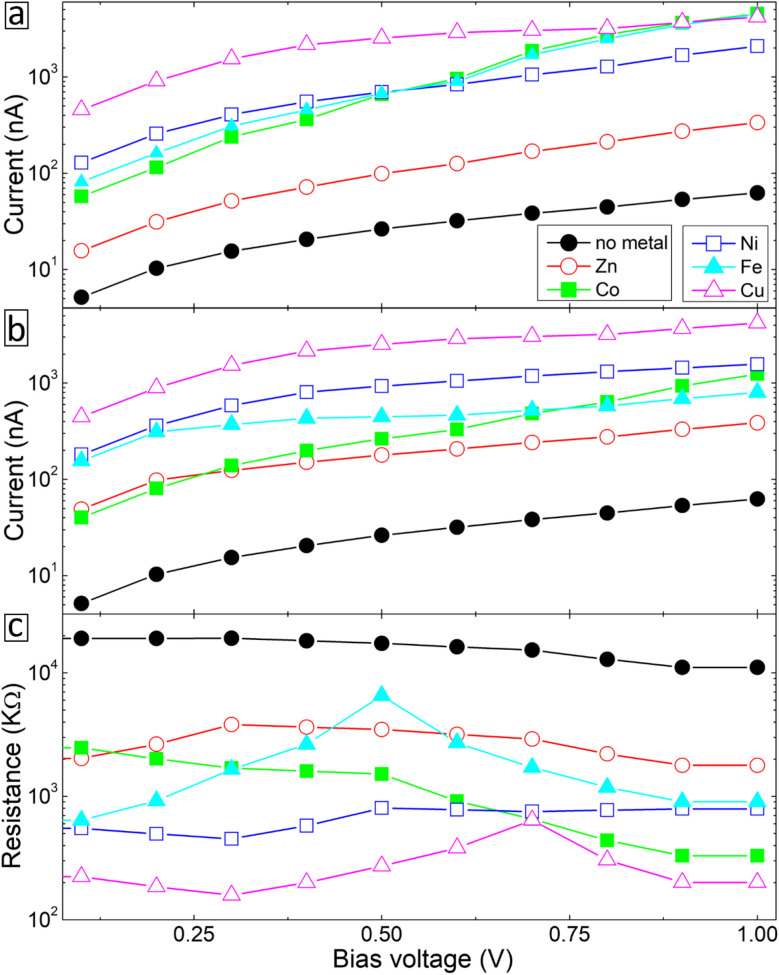
(a and b) *I*–*V* characteristics for spin-up (a) and spin-down (b) electrons through the BPD molecular junction without and with different metal atom embedment. (c) Differential resistance of the considered device geometries calculated for the sum of spin up and spin down electrons.

We start with studying the effect of metal atom inclusion on the electronic transport properties of BPD molecular junctions. The *I*–*V* characteristics of the system for different metal atom inclusions can be seen in Fig. 7. The results are present for spin-up and spin-down electrons, see Fig. 7a and b, respectively. Due to the metallic nature of the junctions finite current is obtained for small bias voltages and the current across the junction increases monotonically with further increasing the applied voltage for all considered systems. Clear spin-dependent transport is obtained in the case of ferroelectric atoms. For example, the current for spin-up electrons can be more than 4 times larger than the current for spin-down electrons in the case of Co atoms (open-blue squares) depending on the applied voltage. Interestingly, regardless of their type, metal atom inclusions increase the current through the junction considerably for any value of the applied voltage. In fact, the current can be increased by more than 2 orders of magnitude in the cases of Cu atom (open-magenta triangles). The effect of the metal atom inclusions on the conductance of the BPD molecular junction is also clearly seen from Fig. 7c, where we plot the differential resistance as a function of the bias voltage for all considered device geometries. The resistance drops at least by a factor of 5 regardless of the type of the metal atoms and applied voltage. Thus, the presence of metal atom inclusions results in significant enhancement of charge transport across the BPD molecular junctions. From an experimental point of view it is seen that all metal ions increase the current through the molecular SAM. The largest current is obtained for the cobalt ions for which we have also obtained the smallest work function (see Table 2).

**Fig. 8:**
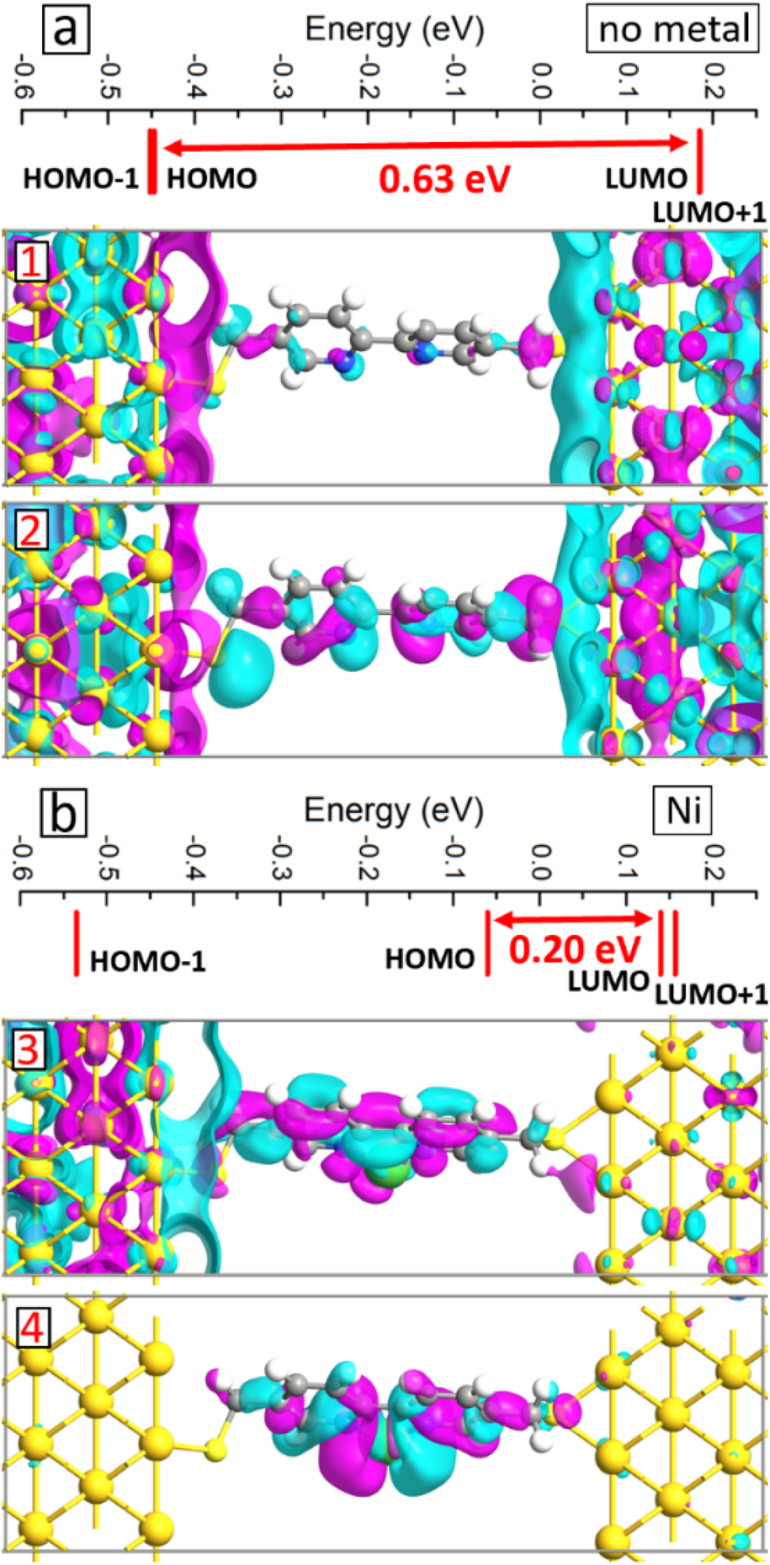
Energies of the eigenstates corresponding to HOMO–1, HOMO, LUMO and LUMO+1 states for the BPD molecular junction without metal atom (a) and with Ni atom inclusion (b). The numbers show the HOMO–LUMO gaps. Panels 1–4 show the isosurface plots of the MPSH states (isovalue ±0.05 Å^−1.5^) corresponding to HOMO (panels 1 and 3) and LUMO (panels 2 and 4) states.

We conducted electronic structure analysis to explain the obtained changes in the *I*–*V* curves due to the metal atom inclusion. We started by analyzing molecular projected MPSH states, which are the eigenstates of the active layer of two-probe devices^34^ and useful to understand the electronic transport at the nanoscale.^21,35^ As typical examples, we present in Fig. 8a and b the MPSH states on the systems without metal atom and with Ni atom, respectively. For the reference system, the HOMO–LUMO gap of 0.63 eV is obtained with the HOMO state localized more near the electrodes, see panel 1 in Fig. 8a, and the LUMO state extended through the junction, see panel 2 in Fig. 8a. The presence of the metal atom makes considerable changes to the electronic structure of the system. First, the HOMO–LUMO gap reduces significantly to 0.2 eV, see Fig. 8b, which has a direct impact on the conductance through the molecular junctions.^36^ Second, all the states near the Fermi level become extended over the junction, see panels 1 and 2 in Fig. 8b. These factors contribute to the current enhancement as we have presented above.

**Fig. 9:**
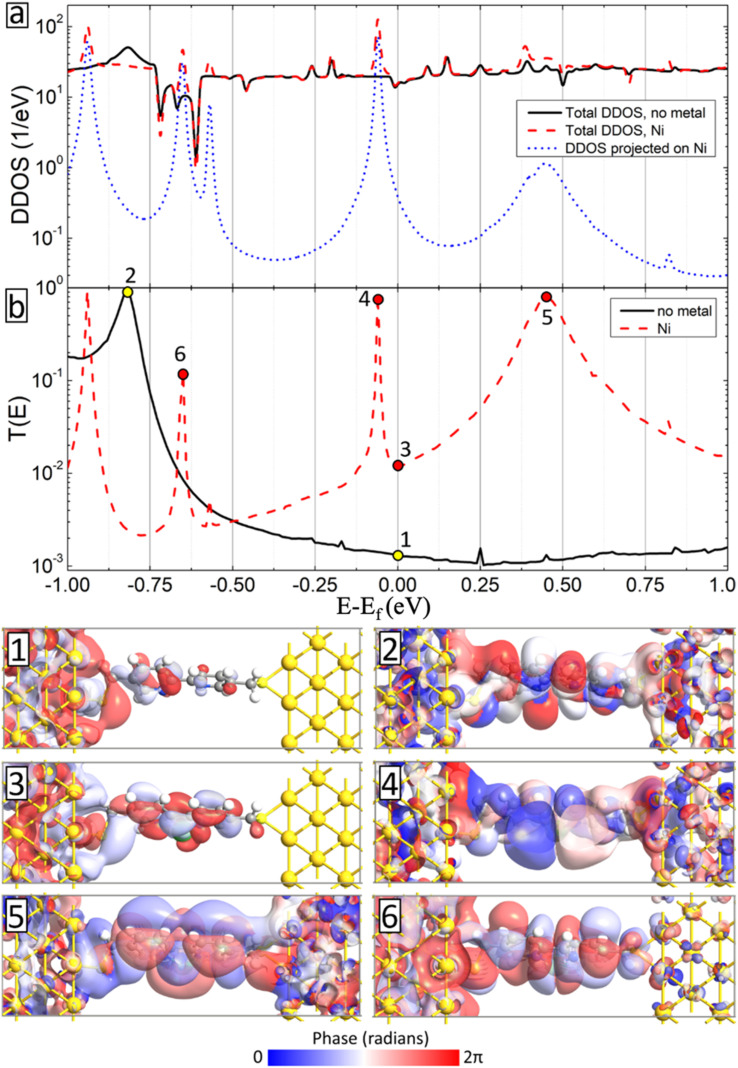
(a) Zero-bias device density of states (DDOS) and (b) transmission spectra as a function of electron energy (zero corresponds to Fermi energy) for the systems without metal atoms (solid-black curves) and with Ni atoms (dashed-red curves). Dotted-blue curve in (a) shows the DDOS of the sample projected on the Ni atom. Panels 1–6 show the isosurface plots (isovalue 0.05 Å^−1.5^ eV^−0.5^) of the transmission eigenstates at electron energies indicated on the transmission curves for the system without metal atom (panels 1 and 2) and with Ni atom (panels 3–6). The results are shown for the eigenstates corresponding to the largest eigenvalue for the given electron energy.

We have also conducted detailed analysis of density of states of the device geometries (DDOS) and their transmission spectra (T(*E*)) for all considered metal atom inclusions. As a typical example, we show in Fig. 9 DDOS (a) and T(*E*) (b) of the BPD junction without (solid-black curves) and with Ni atom (dashed-red curve) at zero voltage bias. These two systems show similar DDOS except additional peaks due to the metal atom. The contribution of the Ni atoms to the DDOS of the system is shown by dotted blue curve in Fig. 9a. Interestingly, each peak on the DDOS contributed by the Ni atom corresponds to pronounced transmission peak indicating the importance of the metal atom embedment to the electronic transport in BPD molecular junctions. Those transmission peaks originate from the extended transmission eigenstates as shown in panels 4–6 in Fig. 9. Because of such pronounced contribution of the metal atom, the transmission spectrum becomes larger as compared to the one for the pristine sample (see solid-black curve in Fig. 9b), especially close to the Fermi level. Panels 1 and 3 show the isosurface plots of the transmission eigenstates at the Fermi level without (panel 1) and with the Ni atom (panel 2). In the former case, we obtained localization of the states near the electrodes and the metal atom contributes to the extension of the state across the junction.

**Fig. 10:**
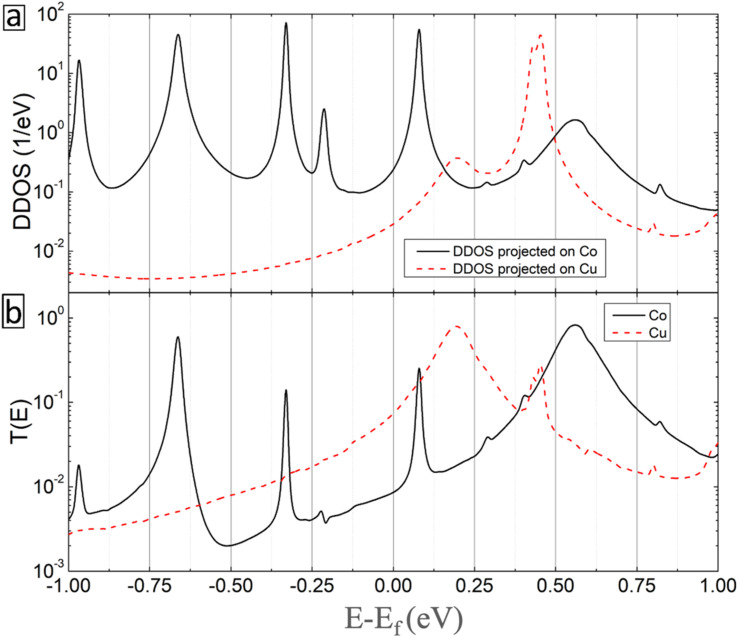
(a) Zero-bias device density of states (DDOS) projected on the metal atoms and (b) transmission spectra as a function of electron energy (zero corresponds to Fermi energy) for the systems with Co (solid-black curves) and Cu (dashed-red curves) atoms.

Fig. 10 shows the DDOS (projected on the metal atoms) and transmission spectra as a function of electron energy for Co (solid-black curves) and Cu (dashed-red curves) inclusions. Clear beaks are obtained in both curves indicating the strong contribution of the metal atoms to the conductivity of the considered molecular junction. Thus, the enhanced current in the BPD molecular junction with embedded metal atoms originates from the extension of the electronic states after metal atom attachment.

**Fig. 11:**
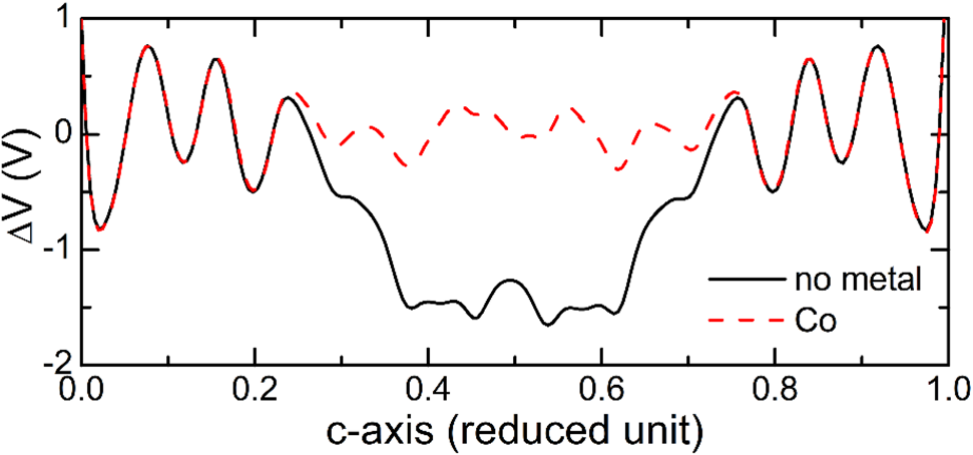
Variations of averaged electrostatic difference potential at zero bias voltage across the BPD junctions without (solid-black curve) and with Co atom (dashed-red curve).

Another factor that affects the electronic transport properties of molecular junctions^37^ is the electrostatic potential profile in the system. To see effect of the metal atom incorporation on the electrostatic potential variations, we plot in Fig. 11 the average electrostatic potential across the BPD junction without (solid-black curve) and with Co atom (dashed-red curve) calculated as the difference between the electrostatic potential of the self-consistent valence charge density (*i.e.*, solution of the Poisson equation) and the one obtained from the superposition of atomic valence densities. It is seen from this figure that the presence of the metal atom strongly reduces the variations of the electrostatic potential inside the junction area. Consequently, the transmission probabilities of the electrons increase because of the reduced scattering of the electrons from the electrostatic potential barriers.

## 3. Conclusions

This study investigates the incorporation of metal ions and their impact on the structural, electronic, and transport characteristics of 5,5′-bis(mercaptomethyl)-2,2′-bipyridine SAMs, employing a combination of experimental techniques and computational simulations. The EGaIn experiments demonstrate that the incorporation of metal ions notably enhances the current through these molecular SAMs. XPS analysis reveals distinct bonding preferences among metal ions; silver ions mainly bond with the sulfur end groups, while other metals tend to bind with the pyridinic regions of the BPD molecules. This bonding pattern leads to a significant reduction in the work function of the molecular SAM–gold complex. Furthermore, XPS data suggest that the interaction with metal ions significantly alters the electronic structure of the pyridinic units in BPD molecules, enhancing electron migration within the molecular system by promoting electron delocalization. Simulations also indicate that attaching metal atoms to the pyridinic units can greatly increase the current through the junction, potentially by up to two orders of magnitude. This increase in current is attributed to structural changes in the system and the emergence of new electronic states near the Fermi level due to the presence of metal atoms. These results are promising for the advancement of molecular device technologies in electronic and energy applications.

## 4. Methods

### 4.1 Sample preparation

The chemicals and solvents were purchased from Sigma-Aldrich and used without further purification. The polycrystalline Au substrate was deposited by magnetron sputtering on silicon (111) wafer. The silicon substrate was ethanol cleaned several times and a Ti buffer layer of 100 nm was first deposited prior to the 300 nm of Au film. Before the SAMs were deposited, the Au surface was cleaned three times with ethanol and dried under N_2_ flow. The SAMs procedure was performed as previously indicated in ref. 38. Briefly, BPD@Au was prepared by using a 1 mM solution of BPD in hexane. The solution was degassed for 15 minutes before immersing the gold substrate, then the solution was heated (60 °C). After that, the gold substrate was washed three times with absolute ethanol and dried with nitrogen gas. The metal intercalation was performed by immersing the prepared BPD SAMs into pre-degassed metal-based salt solution for 1 h under Ar gas bubbling. Then the salt solution was heated for 1 hour at (60 °C). The sample was then rinsed with DI water and dried with N_2_ gas. Surface analysis of the studied SAMs was performed immediately after the sample preparation, to avoid oxidation of the adsorbed molecules.

### 4.2 XPS/UPS measurements

The XPS measurement is performed on an ESCALAB 250Xi thermo fisher platform with a monochromated AlKα source (1486.8 eV). A hemispherical analyzer was used to capture the signal with a take-off angle of 90°. The high-resolution spectra were taken with a pass energy of 20 eV and a step size of 0.1 eV. All measurements were conducted under an ultrahigh vacuum of 10^−10^ mbar at room temperature. The binding energy scale of the obtained core-level spectra was calibrated with respect to the Au 4f 7/2 signal at 84.0 eV. The intensities were normalized to the intensity of Au 4f for direct comparison. UPS measurements were conducted with a He discharge lamp with a beam energy of 21.2 eV (HeI) and the binding energy position was calibrated to the Fermi level.

### 4.3 Transport measurements

The charge transport measurement was performed on a homemade contact angle system with EGaIn top electrode. All junctions were tested with a voltage window of −1 V to 1 V with a step of 0.01 V. We take 10 junctions for every sample and 10 repeated curves per junction to obtain the average results.

### 4.4 Quantum transport calculations

Structural optimizations are conducted using DFT within the generalized gradient approximation of Perdew–Burke–Ernzerhof (PBE) for the exchange–correlation energy^39^ following the optimization procedures described in detail in ref. 37. Grimme's D3 PBE empirical correction^40^ is used to account for non-bonded van der Waals interactions and 5 × 5 × 150 × Monkhorst–Pack^41^*k*-points are taken for Brillouin zone integration. All atoms are described using norm-conserving PseudoDojo pseudo potential with medium basis set.^42^ The convergence criterion for Hellman–Feynman forces was 0.01 eV Å^−1^ and the density mesh cut-off energy was 2176.91 eV in the simulations.

The current–voltage (*I*–*V*) characteristics of the considered systems are calculated using the nonequilibrium Green's function formalism^43^ within the Landauer–Buttiker approach:
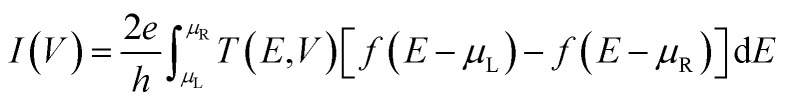
where *T*(*E*,*V*) is the transmission spectrum at a given applied voltage (*V*), *f*(*E*,*E*_F_) is the Fermi–Dirac distribution function and *μ*_L_/*μ*_R_ is the chemical potential of the left/right electrode.

## Data availability

Data is available from the authors upon request.

## Author contributions

H. H. designed the experiment, Y. T., H. H., and M. A. prepared the samples and conducted XPS and transport measurements. G. R. B. conducted quantum transport simulations. All authors contributed to manuscript preparation. H. H. directed the project.

## Conflicts of interest

The authors declare no competing interests.
