# Correction: Nanoarchitectonics of molecular self assembled monolayers by transition metal ion intercalation for enhancement of molecular junction conductivity

**DOI:** 10.1039/d4ra90081b

**Published:** 2024-07-29

**Authors:** Y. Tong, M. Alsalama, G. R. Berdiyorov, Sara Iyad Ahmad, H. Hamoudi

**Affiliations:** a Qatar Environment and Energy Research Institute, Hamad Bin Khalifa University Doha Qatar hhamoudi@hbku.edu.qa hichamhamoudia@gmail.com; b HBKU Core Labs, Hamad Bin Khalifa University Doha Qatar

## Abstract

Correction for ‘Nanoarchitectonics of molecular self assembled monolayers by transition metal ion intercalation for enhancement of molecular junction conductivity’ by Y. Tong *et al.*, *RSC Adv.*, 2024, **14**, 21597–21607, https://doi.org/10.1039/D4RA02950J.

The authors regret the omission of a funding acknowledgement in the original article. This acknowledgement is given below.

The authors would like to acknowledge open access funding provided by the Qatar National Library.

The Royal Society of Chemistry apologises for these errors and any consequent inconvenience to authors and readers.

